# Microbial diversity in various types of paper mill sludge: identification of enzyme activities with potential industrial applications

**DOI:** 10.1186/s40064-016-3147-8

**Published:** 2016-09-06

**Authors:** Manel Ghribi, Fatma Meddeb-Mouelhi, Marc Beauregard

**Affiliations:** 1 CRML, Centre de Recherche sur les Matériaux Lignocellulosiques, Université du Québec à Trois-Rivières, C.P. 500, Trois-Rivières, QC G9A 5H7 Canada; 2 PROTEO, Université Laval, Quebec, QC G1V 4G2 Canada

**Keywords:** Bacteria, Enzymes, Bioplastic, Pulping liquors, Dyes

## Abstract

This study is the first comprehensive investigation of enzyme-producing bacteria isolated from four sludge samples (primary, secondary, press and machine) collected in a Kraft paper mill. Overall, 41 strains encompassing 11 different genera were identified by 16S rRNA gene analysis and biochemical testing. Both biodiversity and enzymatic activities were correlated with sludge composition. Press sludge hosted the largest variety of bacterial strains and enzymatic activities, which included hydrolytic enzymes and ligninolytic enzymes. In contrast, strains isolated from secondary sludge were devoid of several enzymatic activities. Most strains were found to metabolize Kraft liquor at its alkaline pH and to decolorize industrial lignin-mimicking dyes. Resistance to lignin or the ability to metabolize this substrate is a prerequisite to survival in any paper mill sludge type. We demonstrate here that the bacterial strains found in a typical Kraft paper mill represent a source of potential novel enzymes for both industrial applications and bioremediation.

**Electronic supplementary material:**

The online version of this article (doi:10.1186/s40064-016-3147-8) contains supplementary material, which is available to authorized users.

## Background

Industrial enzymes are at the heart of green chemistry as illustrated by a global market value that reached 5 G$ in 2013 which is expected to climb to 7 G$ by 2018, a compound annual growth rate of 8.2 % over 5 years (Global Markets for Enzymes in Industrial Applications; Nigam and Pandey 2009). While all living organism produce enzymes, microorganisms have been and are increasingly being used as cell factory for enzyme production, and as a source for various chemicals (therapeutics, bioethanol and bioplastics to name few) (Adrio and Demain 2005; Ai et al. 2003; Charbonneau et al. 2011; Demain and Adrio 2008; Paye et al. 2015; Rehman et al. 2007). Currently, most industrial enzymes originate from microorganisms like *Bacillus*, *Aspergillus and Trichoderma* (Apparao and Krishnaswamy 2015; Arora and Sandhu 1985; Bengtsson et al. 2006; Demain and Adrio 2008; Kubicek et al. 2001; Liu et al. 2013; Pandey et al. 1999). These biocatalysts have found applications in various fields that depend on hydrolytic, ligninolytic and biosynthetic processes, to name a few (Barr and Steven 1994; Braunegg et al. 2004; Chandra et al. 2011; Martínez et al. 2005; Nigam 2013).

Hydrolases (or hydrolytic enzymes) are the largest group of enzymes on the market and are used in detergents, pharmaceuticals, therapeutics, textiles, baking, biofuel and pulp and paper (Bengtsson et al. 2006; Crecchio et al. 1995; Eriksson and Kirk 1994; Kirk et al. 2002). Hydrolases include carboxyl ester hydrolases (lipases, esterases), glycosylases (cellulases, xylanases and amylases) and proteases that hydrolyze lipids, sugar polymers and proteins, respectively. Application of ligninolytic enzymes [lignin peroxidase (LiP), manganese peroxidase (MnP) and laccase (Lac)] is a growing sector of industrial enzymology. In recent years ligninolytic enzymes have been shown to be effective in industrial applications, including bio-remediation, pollution control and treatment of industrial effluents containing recalcitrant and hazardous chemicals such as textile dyes and or lignin mimicking dyes, phenols and other xenobiotic (Bandounas et al. 2011; Brahimi Horn et al. 1992; Chandra et al. 2011). This group of enzymes is also used in the pulp and paper industry for pre-treatment of wood pulp, for bio-bleaching and bio-pulping (Eriksson and Kirk 1994; Huang et al. 2007).

In addition to their use as enzyme production factories, microorganisms have attracted a growing interest for their biosynthetic pathways, including those leading to the synthesis of biodegradable plastics, such as polyhydroxyalkanoates (PHAs) (Singh et al. 2009; Verlinden et al. 2007). PHA polymer is synthesized and accumulated by bacteria as means for storing carbon and energy during unbalanced growth conditions (Chen 2009). Applications for PHA have been developed in various fields such as bioplastics, fine chemicals, implant biomaterials, medicines and biofuels (Gomaa 2014; Nathalie et al. 2015).

Screening bacteria for industrially useful enzymes is now a key endeavor in industrial biotechnology. Bacteria and bacterial enzymes are becoming much more important for the treatment of lignocellulosic materials, as some outperforming commercial fungal extracts for the hydrolysis of cellulose and hemicellulose fibers (Hardiman et al. 2010; Maki et al. 2011; Singh and Prabhu 1986; Zhou and Ingram 2000). Bacteria generally inhabit ecological niches characterized by specific attributes such as pH, temperature, specific carbon or substrate availability, presence of salt and other chemical factors (solvent, inhibitors, toxicants, oxygen, etc.). Such attributes determine the array of enzymes and metabolic routes necessary for survival and are an important aspect of screening. In our laboratory we have taken advantage of this relationship between enzymes and niche attributes for identification of novel strains and enzymes from compost (Charbonneau et al. 2011).

The wastewaters or sludge generated from paper making processes are enriched with various fiber wood compounds such as lignin, carbohydrate polymers (cellulose and hemicellulose) and other extractives (lipids and others) in addition to some potentially toxic compounds such as chlorinated organics, resin acids, heavy metals and others (Abhay et al. 2007; CANMET Energy Technology Centre 2005; Karn et al. 2010; Kuhad et al. 1997). The sludge environments could be effective incubators of a wide variety of resistant, adapted bacteria and should offer an ideal source of enzymes suitable for related industrial applications. Consequently, investigations on paper mill sludge microflora have been previously reported. Studies by Maki et al. (2011), Karn et al. (2013, 2010), and Desjardins and Beaulieu (2003) showed that paper mill sludge is a great incubator and host for a variety of microorganisms. Such studies have focused on a precise type of process, used a single sludge type, focused on one particular enzyme, and revealed limited information on sludge microflora. Various types of sludge are produced at different steps along the paper making process, in order to manage waste effluents. To our knowledge, the biodiversity of individual sludge types found in a Kraft paper mill has yet to be studied. Here, we report an exhaustive characterization of the microflora existing in three types of paper mill sludge and one environmental sample obtained from an Eastern Canadian Kraft paper mill. We established a screening for a wide range of extracellular enzymes produced by bacteria found in these sludge samples. We also identified bacterial strains that have various potential applications in pulp and paper, pollution control, bioremediation and bioplastic production. Sludge sampled at different production stages revealed important differences in bacterial strains and enzyme production despite being sampled at the same facility.

## Methods

### Paper mill sludge sampling and characteristics

Four sludge samples were collected from a paper mill at Trois-Rivières (Québec, Canada). Samples were collected in plastic bags under aseptic conditions and marked according to their source and location. The temperature was measured on site of collection. The collected samples were transported to the laboratory for the isolation of sludge bacteria. All sludge samples were stored at 4 °C, used and processed within 48 h. Sludge descriptions and characteristics were reported in Table 1.

**Table 1:** Paper mill’s sludge characteristics

Sample	Origin	Color	pH	T °C
Primary sludge (IS)	Discharges from pulp processing which are rich in cellulose-fiber and undergo settling, solid state	Dark brown	7.2	37
Secondary sludge (IIS)	Decantation of primary sludge, undergo another physical settling, solid state	Dark brown	7.0	30
Press sludge (PS)	Mixture of secondary and primary sludge, solid state	Dark brown	3.0	35
Machine samples (MS)	Environmental samples found around machine in the mill, solid state	Light brown	7.2	37

Primary sludge (IS) recovered at the first step of clarification is usually obtained by sedimentation. IS sludge is dark brown in color, having a pH and temperature of 7.2 and 37 °C respectively. The secondary sludge (IIS) is generated in the clarifier of the biological unit used for wastewater treatment. IIS volumes are lower than those corresponding to the primary sludge, since most of the heavy, fibrous or inorganic solids are removed in the primary clarifier (CANMET Energy Technology Centre 2005; Integrated Pollution Prevention and Control (IPPC) 2001). IIS temperature and pH are slightly less than found in IS. Press sludge (PS) is a mixture of primary sludge and secondary sludge following acid treatment, and its pH and temperature are around 3 and 30 °C respectively. PS is the richest in carbonic matter due to the fact it is a concentrated mixture of two different kinds of sludges (primary and secondary). An additional sample used for this study was an environmental sample collected from organic deposits (dirt) around paper mill machines (MS). This sludge was light brown, had a neutral pH and was of moderate temperature (ambient to few degrees above ambient temperature).

### Isolation and growth of bacteria from sludge samples

To isolate the bacteria contained within each sludge sample, a 1.0 g sub-sample was diluted in 100 ml of saline (0.85 % NaCl), thoroughly mixed by vortexing, and incubated for 1 h at 50 °C. After which the solution was filtered through a sterile filter paper to remove all solids. A 200 µl volume of filtrate from each sludge sample was spread on minimum media (Mm) agar plate (0.1 % NaNO_3_, 0.1 % K_2_HPO_4_, 0.05 % MgSO_4_, 0.1 % KCl, 0.05 % yeast extract and 1.5 % Bacto agar) supplemented with 1 % of the corresponding sludge with the pH of media adjusted to 7.0, the most suitable pH to enhance and promote the growth of large number of bacteria. Plates were incubated at 37 °C for 48 h. Following incubation, colonies having macroscopically different morphologies were picked from each plate and re-streaked on LB agar media to obtain pure cultures. For long term storage, isolates were grown overnight in 10 ml LB medium (pH 7.0) under agitation at 230 rpm and 1 ml of each overnight culture stored as suspension in 30 % (v/v) glycerol at −80 °C.

### Morphological identification and physiological characterization of the different strains

For each isolate the colony characteristics (shape, size, pigmentation, texture, and opacity), cell morphology, and Gram stain reaction were determined. Catalase and oxidase activities were determined as described by Meddeb-Mouelhi et al. (2015).

### Screening of hydrolytic enzyme-producing bacteria

Single colonies from fresh LB agar plates were patched onto minimal media (Mm) agar plates (0.1 % NaNO_3_, 0.1 % K_2_HPO_4_, 0.05 % MgSO_4_, 0.1 % KCl, 0.05 % yeast extract and 1.5 % Bacto agar) pH 7.0, incubated at 37 °C, and supplemented with the appropriate substrate for extracellular hydrolytic enzyme detection (Charbonneau et al. 2011). For the detection of extracellular enzyme activity, buffered Mm was supplemented with the following substrates: glyceryl tributyrate 1 % (v/v) for esterase activity; olive oil 1 % (v/v) containing Rhodamine B 0.001 % (w/v) for detection of lipolytic activity (Fariha et al. 2010; Gupta et al. 2004; Jaeger and Reetz 1998); carboxymethyl cellulose (CMC) 0.5 % (w/v) for CMCase activity and Phosphoric Acid Swollen Avicel (PASA) 0.1 % (w/v) for PASAase activity (Kubicek et al. 2009; Singh and Prabhu 1986; Wang et al. 2010). Remazol Brillant Blue-xylan (RBB-xylan) from beechwood 0.5 % (w/v) was used for xylanase activity. Starch 1 % (w/v) was used for amylase activity (Meddeb-Mouelhi et al. 2015; Nigam and Singh 1995) and 1 % of casein (w/v) was used for protease activity (Nigam and Pandey 2009; Nigam et al. 2013; Van Dyke 2004). Lipolytic activity was visualized by illuminating the plate with UV light (302 nm) which results in an orange fluorescence detected at 535 nm around colony producing lipase (Patil et al. 2011; Sharyo 1993). Extracellular esterase activity was observed by appearance of a clear zone around the colonies (Kuhad et al. 1997; Meddeb-Mouelhi et al. 2015). CMCase and PASAase activities were detected by staining the plates using 0.2 % Congo red for 15 min and then de-stained by washing twice with 1 M NaCl for 15 min (Meddeb-Mouelhi et al. 2015). Xylanase positives clones were identified by a clear zone around the colonies on RBB-xylan plate (Meddeb-Mouelhi et al. 2015). Industrial enzymes (lipases, cellulases, xylanases and esterases purchased from Buckman North America) were used as positive controls.

### Detection of ligninolytic enzymes

Laccase activity was detected using Mm agar plates at pH 5.0, supplemented with 6 mM ABTS (3-ethylbenzothiazoline-6-sulphonic acid) and 100 mM of CuSO_4_. After incubation at 37 °C, a positive reaction was indicated by the appearance of a green or violet color surrounding the bacterial colony (Arora and Sandhu 1985; D’Souza et al. 2009). LiP activity was screened for using Mm agar plates supplemented with 2 mM hydrogen peroxide (H_2_O_2_) (30 %) and 0.02 % Azure B at 37 °C pH 7.0 as described by Arora and Sandhu (1985). Isolates displaying LiP activity were detected by the appearance of clear halo around the colonies (Oyadomari et al. 2003; Singh and Prabhu 1986; Wongwilaiwalin et al. 2010). The detection of MnP activity was carried out as previously described by Orth et al. (1993) using Boyd and Kohlmeyer (B&K) agar plates (0.1 % yeast extract, 0.2 % peptone, 1 % dextrose, 1 mM CuSO_4_, and 1.5 % Bacto agar) supplemented with 0.1 % magnesium sulfate and 0.1 % phenol red (w/v), neutralized at pH 7.0 and incubated at 37 °C. A positive reaction elicited by a yellow zone surrounding the colony (Hofrichter 2002; Oliveira et al. 2009; Orth et al. 1993).

### Bacteria growth and agar plate decolorization of Kraft pulping liquors

In this experiment, three different Kraft pulping liquors (black, green and white), obtained from an Eastern Canadian paper mill were used. Colonies isolated from the same paper mill as described above were patched onto minimal media (Mm) agar plates (0.1 % NaNO_3_, 0.1 % K_2_HPO_4_, 0.05 % MgSO_4_, 0.1 % KCl, 0.05 % yeast extract and 1.5 % Bacto agar) supplemented with 5 % of each liquor (Negrão et al. 2015). All the tests were done at pH 12.0, the closest to real pH of pulping liquors, and incubated at 37 °C. Both the rate of colony growth and features (appearance and color) were recorded over the 5 days of incubation.

### Bacteria decolorization of lignin-mimicking dyes

Four different lignin-mimicking dyes, methyl blue (MB), methyl green (MG), remazol brilliant blue (RBB) and Chicago sky blue (CSB) were used in this study. Dye de-colorization by the isolates was carried out according to Kiiskinen et al. (2004). Bacteria were picked onto Mm or LB agar plates (pH 7.0) supplemented with 0, 05 % (w/v) of the dye. The respective dye activity was visualized within 24–48 h of incubation at 37 °C. The appearance of a decolorized halo surrounding the colony indicated the dye degradation by the bacteria. The color of cell colony was also visually inspected to establish whether the dye had been absorbed by the colony rather than being degraded (Daizong et al. 2014; Wesenberg et al. 2003).

### Identification of PHA producing bacteria

PHA-producing bacteria were identified with two different staining methods using Sudan Black B (SBB) and Nile Blue A (NBA). For the first method, bacteria were grown on PHA Detection (PD) liquid medium (0.2 % (NH_4_)_2_SO_4_, 1.33 % KH_2_PO_4_, 0.13 % MgSO_4_, 0.17 % citric acid, 2 % of glucose as carbon source and 10 mL/L of trace element solution) as described by Naheed and Jamil (2014). Media culture pH was adjusted to 7.0. After overnight incubation, 200 µl bacteria culture were re-streaked on PHA Detection Agar (PDA) as described in Gomaa (2014). After 24 h incubation at 37 °C, PHA accumulation in cells was detected by SBB staining method. For this, 0.002 % SBB solution in 100 % ethanol was gently spread over the plates completely soaking them, and then the plate incubated at room temperature for 30–60 min (Rieger et al. 2002). The solution was then discarded and the plate rinsed gently with 100 % ethanol. Colonies unable to incorporate SBB appeared white, while PHA producers appeared bluish black. All isolated which appeared to produce PHA were confirmed by NBA (Nile Blue A) staining according to Kitamura and Doi (1994).

### PCR amplification of 16S rRNA gene and sequencing

Genomic DNA was isolated from selected organisms by 2 heat shock cycles (15 min at −80 °C and 10 min at 100 °C). Amplification of 16S rRNA gene was performed using the universal primers 1522R (sequence 5′–3′: AAGGAGGTGATCCANCCGCA), and 27F (sequence 5′–3′: AGAGTTTGATCMTGGCTCAG). The PCR products were separated on a 0.8 % agarose low melting gel and the band corresponding to 1.5 kb 16S rRNA gene was purified using Qiagen MinElute PCR purification kit and then quantified. Purified PCR products were sequenced on both stands with an ABI Prism 3100 automatic sequencer at the Biomolecular analysis platform (University Laval, QC). Analysis of 16S rDNA sequences were performed using Clone Manager professional 7.0 (Sci Ed Centra). The resulting sequences searched against the nucleotide collection at Genbank database using BLAST with the BLAST algorithm using the non-redundant nucleotide database GenBank via the National Center for Biotechnology Information (NCBI) website (Kumar et al. 2002; Meddeb-Mouelhi et al. 2015).

## Results and discussion

### Identification of bacterial strains from paper mill sludge samples

Forty-one strains were tentatively identified based on their 16S rRNA gene sequences (Table 2). The majority were closely related to previously identified species (97–99 % DNA sequence similarity) and are identified as such. Others were more distantly related (86–95 % DNA sequence similarity). The latter strains could represent new strains, different from the closest relatives listed in Table 2. Overall the strains aligned primarily within the phyla *Firmicutes* (61 %) and *Proteobacteria* (39 %), and encompassed eleven different genera. *Bacillus* and *Paenibacillus* species accounted for twenty-five of the total strains, with the remainder related to species in the genera *Aeromonas*, *Xanthomonas*, *Pseudoxanthomonas*, *Serratia*, *Rahnella*, *Pantoea*, *Citobacter*, *Klebsiella* and *Raoultella* (Table 2). One possible limitation of this work is that both the isolation media and approach utilized may have affected the final distribution of strains across these samples. Specifically, there remains the possibility of additional anaerobic and uncultivable bacterial strains associated with these samples.

**Table 2:** Genetic identification of screened strains from paper mill sludges

Strains	Phyllum	Genetic identification (closest relative)	% sequence identity
PS1	*Firmicutes*	*Bacillus amylolequifaciens*	92
PS2	*Proteobacteria*	*Aeromonas salmonicida*	99
PS3	*Firmicutes*	*Paenibacillus* sp.	92
PS4	*Firmicutes*	*Paenibacilus* sp.	92
PS4.2	*Proteobacteria*	*Klebsiella pneumonia*	89
PS5	*Firmicutes*	*Bacillus subtilis*	94
PS6	*Firmicutes*	*Bacillus subtilis*	*93*
PS7	*Firmicutes*	*Bacillus* sp.	89
PS8	*Firmicutes*	*Bacillus subtilis*	86
PS9	*Firmicutes*	*Bacillus subtilis*	89
PS10	*Firmicutes*	*Bacillus subtilis*	99
PS11	*Firmicutes*	*Bacillus subtilis*	99
PS12	*Firmicutes*	*Paenibacillus* sp.	99
PS13	*Firmicutes*	*Bacillus tequilensis*	89
PS14	*Firmicutes*	*Bacillus* sp.	92
PS15	*Firmicutes*	*Bacillus thuringiensis*	98
PS16	*Proteobacteria*	*Citrobacter* sp.	98
PS17	*Firmicutes*	*Bacillus* sp.	98
PS18	*Firmicutes*	*Bacillus subtilis*	98
PS19	*Firmicutes*	*Bacillus cereus*	98
PS20	*Firmicutes*	*Bacillus thuringiensis*	98
PS21	*Firmicutes*	*Bacillus subtilis*	89
PS22	*Firmicutes*	*Bacillus subtilis*	86
MS1	*Proteobacteria*	*Klebsiella* sp.	97
MS2	*Proteobacteria*	*Xanthomonas* sp.	99
MS3	*Proteobacteria*	*Pantoea* sp.	98
MS4	*Firmicutes*	*Paenibacillus taichungensis*	95
MS5	*Proteobacteria*	*Serratia* sp.	98
MS6	*Proteobacteria*	*Serratia* sp.	98
MS7	*Proteobacteria*	*Pseudoxanthomonas taiwanensis*	98
MS8	*Proteobacteria*	*Raoultella terrigena*	98
MS9	*Proteobacteria*	*Raoultella terrigena*	98
MS10	*Proteobacteria*	*Serratia* sp.	98
MS11	*Firmicutes*	*Bacillus subtilis*	98
IS1	*Proteobacteria*	*Rahnella aquatilis*	98
IS2	*Proteobacteria*	*Rahnella* sp.	95
IS3	*Firmicutes*	*Bacillus* sp.	98
IS4	*Firmicutes*	*Bacillus cereus*	98
IIS1	*Proteobacteria*	*Aeromonas* sp.	97
IIS2	*Firmicutes*	*Paenibacillus* sp.	98
IIS3	*Proteobacteria*	*Aeromonas* sp.	98

Phyllum and genetic identification of bacteria are conventionally written in italics

Species within the genera *Bacillus, Paenibacillus* and *Xanthomonas* have been found in paper mills in the USA, Finland, New Zealand and Canada to name a few (Beauchamp et al. 2006; Chiellini et al. 2014; Desjardins and Beaulieu 2003; Harju-Jeanty and Vaatanen 1984; Kämpfer et al. 2012; Schallmey et al. 2004; Martin 1988; Niemelä and Väätänen 1982). We found that the dominant genus in our mill was *Bacillus,* confirming previous biodiversity studies in pulp and slime samples from different Canadian paper mills (Desjardins and Beaulieu 2003; Maki et al. 2011). We also isolated species in the genera *Paenibacillus, Raoultella, Serratia, Klebsiella* and *Xanthomonas* which have not previously been reported in earlier paper mill biodiversity studies. Differences in microflora also occurred among the sludge samples. Twenty-three strains originated from PS sludge, eleven from MS, and much fewer from the IS and IIS samples (4 and 3 strains respectively).

### Morphological and physiological characterization of strains from paper mill sludge samples

Strains were further characterized on the basis of colony morphology (colour, shape, margin, elevation and texture), cell morphology, Gram reaction, oxidase, and catalase activities (Additional file 1: Table S1). Twenty-one strains displayed oxidase and catalase activities, sixteen showed only catalase, and four strains only oxidase activity. Thirty-one strains were bacilli (rod-shaped) and stained Gram-positive. Ten strains were cocci and stained Gram-negative (see Additional file 1: Table S1). The majority of bacteria isolated from IS and PS were Gram-positive, whereas the majority of MS and IIS were Gram-negative.

All strains were tested for their ability to grow at various pH and temperatures. Among the strains, twenty-one grew at pH ranging from 6 to 10, while twelve strains grew at pH ranging from 7 to 10. The remaining eight strains grew only at neutral pH (Additional file 1: Table S2). Strains were also tested for their ability to grow at 50 °C. All of the strains isolated from PS grew at 50 °C at pH values up to 10, with the exception of strains PS5, PS7, PS12, PS15 and PS17 which grew only at neutral pH (Additional file 1: Table S2). Strains isolated from other environmental samples MS, IS and IIS were generally unable to grow at 50 °C, with the exception of two strains (MS1 and MS2).

While various *Bacillus* spp. were isolated from all of the sludge samples, they dominated the cultivable community from PS. The finding is not unexpected, as species within this genus have been detected in a wide range of environmental screening studies (Desjardins and Beaulieu 2003; Vaisanen et al. 1991). Furthermore, sludge samples contain a rich content of polymers (cellulose and hemicellulose), and PS being a combinational end-product is more concentrated than the IS and IIS sludge used to produce it and accordingly, revealed to be the most prolific medium regarding biodiversity.

### Hydrolytic activities associated with the bacteria screened from paper mill sludge

All strains were screened for seven different hydrolytic activities (CMCase, PASAase, xylanase, esterase, lipase, protease and amylase) (Table 3). Overall, twenty-one strains expressed extracellular cellulase (CMCase and/or PASAase). Among these, fourteen showed a preference for CMC, whereas four others preferred amorphous cellulose-rich PASA, and three strains PS1, PS5, and PS10 hydrolysed both CMC and PASA. The remaining twenty strains were xylanase positive and were primarily related to species in the genus *Bacillus*. Only four strains displayed both cellulase and xylanase activity. These were IIS1, IS3, PS21 and MS10, and were identified as *Aeromonas* sp., *Bacillus* sp., *Bacillus subtilis* and *Serratia* sp. respectively.

**Table 3:** Enzymatic activities of screened bacterial strains on agar plate with relevant substrate at 37 °C and pH 7

Strains	Genetic identification	Hydrolytic enzymes							Ligninolytic enzymes		
		Est	Lip	CMC	PASA	Xyl	Amyl	Prot	LiP	MnP	Lac*
PS1	*Bacillus amylolequifaciens*	+	−	+	+	−	−	+	−	+	+
PS2	*Aeromonas salmonicida*	+	−	+	−	−	+	+	−	−	−
PS3	*Paenibacillus* sp.	+	+	+	−	−	−	−	+	−	+
PS4	*Paenibacilus* sp.	+	−	+	−	−	−	−	−	−	−
PS4.2	*Klebsiella pneumonia*	+	−	+	−	−	−	+	+	+	+
PS5	*Bacillus subtilis*	+	−	+	+	−	+	+	+	−	−
PS6	*Bacillus subtilis*	+	−	+	−	−	+	+	−	+	−
PS7	*Bacillus* sp.	+	+	+	−	−	−	−	−	−	−
PS8	*Bacillus subtilis*	+	+	+	−	−	−	−	+	−	−
PS9	*Bacillus subtilis*	+	−	+	−	−	+	+	−	−	−
PS10	*Bacillus subtilis*	+	−	+	+	−	−	+	+	−	−
PS11	*Bacillus subtilis*	+	+	+	−	−	−	+	+	−	−
PS12	*Paenibacillus* sp.	+	+	+	−	−	−	−	+	+	+
PS13	*Bacillus tequilensis*	+	+	+	−	−	+	+	−	−	−
PS14	*Bacillus* sp.	+	+	−	−	+	+	+	−	+	−
PS15	*Bacillus thuringiensis*	−	−	−	−	+	−	−	−	−	−
PS16	*Citrobacter* sp.	+	−	−	−	+	+	−	−	−	−
PS17	*Bacillus* sp.	−	−	−	−	+	−	−	−	−	−
PS18	*Bacillus subtilis*	+	−	−	−	+	+	+	+	−	+
PS19	*Pantoea* sp.	+	−	−	−	+	−	−	−	−	−
PS20	*Bacillus thuringiensis*	+	+	−	−	+	+	+	+	−	+
PS21	*Bacillus subtilis*	+	−	−	+	+	−	−	+	−	−
PS22	*Bacillus subtilis*	+	+	−	−	+	+	+	−	+	+
MS1	*Klebsiella* sp.	+	−	+					+		+
MS2	*Xanthomonas* sp.	+	−	+	−	−	−	−	−	−	−
MS3	*Bacillus cereus*	−	−	−	−	+	+	+	+	+	−
MS4	*Paenibacillus taichungensis*	−	−	−	−	+	−	−	+	+	−
MS5	*Serratia* sp.	−	−	−	−	+	−	+	−	+	−
MS6	*Serratia* sp.	−	−	−	−	+	−	−	−	−	+
MS7	*Pseudoxanthomonas taiwanensis*	−	−	−	−	+	+	−	−	−	−
MS8	*Raoultella terrigena*	−	−	−	−	+	−	−	−	−	−
MS9	*Raoultella terrigena*	+	−	−	−	+	−	+	+	+	+
MS10	*Serratia* sp.	+	−	−	+	+	−	−	−	−	−
MS11	*Bacillus subtilis*	−	−	−	−	+	−	+−	−	−	−
IS1	*Rahnella aquatilis*	+	−	+	−	−	−	−	−	−	−
IS2	*Rahnella* sp.	−	−	+	−	−	−	−	+	−	+
IS3	*Bacillus* sp.	−	−	−	+	+	+	+	−	−	+
IS4	*Bacillus cereus*	−	−	−	−	+	−	−	−	−	−
IIS1	*Aeromonas* sp.	+	−	−	+	+	+	−	−	−	−
IIS2	*Paenibacillus* sp.	+	−	−	−	+	−	−	−	−	−
IIS3	*Aeromonas* sp.	+	−	−	−	+	−	−	−	−	−

*Est* esterase, *Lip* lipase, *CMC* CMCase, *PASA* PASAase, *Xyl* xylanase, *Amyl* amylase, *LiP* lignin peroxidases, *MnP* manganese peroxidase, *Lac* laccase, *Prot* protease

* Test done at pH5 for 7 days incubation

Bacteria isolated from PS demonstrated either cellulose or xylanase activity, whereas those from MS strains were predominantly xylanolytic, with a few showing cellulase activity. Strains isolated from IS and IIS were better producers of cellulase than xylanase. Higher numbers of xylanase producers from MS likely reflect the abundance of xylan fibers in this particular environmental sample (Raj et al. 2013). All strains isolated from PS displayed extracellular esterase activity when grown on tributyrate agar plate except for strains PS15 and PS17. Nine strains (PS3, PS7, PS8, PS11, PS12, PS13, PS14, PS20 and PS22) identified as various *Bacillus* and *Paenibacillus* strains displayed both lipolytic activities (esterase and lipase). Fourteen strains isolated from PS showed no secreted lipase activity. Four strains isolated from samples taken from machinery (MS1, MS2, MS9 and MS10), three from IIS (IIS1, IIS2 and IIS3) and one from IS (IS1) were also esterase positive. No lipase activity was found in the strains isolated from MS, IS and IIS samples as shown in Table 3. The secretion of esterase observed here appears to be a consequence of PS particular composition, which includes wax esters. Most IIS and PS strains showed esterase activities, unlike those from MS and IS. Lack of lipolytic activities in MS sludge can be explained by its low content in extractible lipidous material.

Screening using agar plates supplemented with starch and casein revealed that 9 strains from PS (PS2, PS5, PS6, PS9, PS13, PS14, PS18, PS20 and PS22), one from MS (MS3) and one from IS (IS3) displayed both amylase and protease activities (Table 3). These strains were predominantly related to *Bacillus*. Twenty strains from various genera (*Bacillus, Paenibacillus, Aeromonas, Xanthomonas, Serratia, Rahnella, Pantoea, Klebsiella and Raoultella*) were amylase and protease negative. We also found that only three strains (MS7, IIS1 and PS16) were amylase positive, and seven (PS1, PS4.2, PS10, PS11, MS5, MS9 and MS11) were protease positive. The finding that most strains produced esterase activity (twenty-nine), cellulase (twenty-two) and xylanase (twenty-three) was no real surprise. In fact, the majority of available substrate in this Kraft paper mill are triglycerides, wax esters, cellulose and hemicelluloses fibers (xylan) (Pervaiz and Sai 2012).

Isolates in the genus *Bacillus* are known for their diversity of enzymatic activities. The capacity of certain *Bacillus* strains to both produce and secrete a large array of extracellular enzymes made them among the most important industrial bacteria producing for enzymes [contributing to about 50 % of the total enzyme market (Adrio and Demain 2005; Fisher et al. 2014; Houde et al. 2004)].^1^ In regard to all of the hydrolytic activities (hydrolase, glycosidase and protease), five strains isolated from PS displayed five of the seven hydrolytic activities which we tested for. These strains included *Bacillus subtilis* PS5, *Bacillus tequilensis* PS13*, Bacillus* sp. PS14, *Bacillus thuringiensis* PS20 and *Bacillus subtilis* PS22 (Table 3). Overall, the variability in strain enzymatic profiles likely reflects differences in sludge composition or conditions, and adaptation of the microflora to each specific environment. Again, both the detection methods and substrates used for detection of enzymatic activities can impact the observed enzymatic profiles. For instance in the case of enzymes that are not secreted, they would not likely be detected. Moreover, the limited number of substrates used here will not reveal all of the potential enzymatic activities present in any given sample.

### Detection of ligninolytic enzymes

To further characterize biodiversity we examined the ligninolytic potential of these strains. Ligninolytic enzymatic activities screened for including laccase (Lac), lignin peroxidase (LiP) and manganese peroxidase (MnP) (Hofrichter 2002; Kumar et al. 2008; Martínez et al. 2005). Twenty-two strains expressed at least one extracellular ligninolytic enzymatic activity (Table 3). Three strains (*Paenibacillus* sp. PS12, *Klebsiella pneumonia* PS4.2 and *Raoultella terrigena* MS9) produced all three activities, whereas nineteen strains showed none.

Strains isolated from PS sludge demonstrated the greatest number of extracellular ligninolytic activities compared to MS, IS and IIS where these activities were not detected. This may be due to both compositional and environmental differences among these sludges. In fact, the former contains organic matter such as cellulose, lignin and wood extracts (Negrão et al. 2015; Oyadomari et al. 2003) which make this sludge an optimal niche for ligninolytic enzymes producing bacteria. *Paenibacillus* strains have previously been identified in paper mill samples; however unlike strains PS12, PS4, and IIS2, no enzymatic activity profiles were reported before (Kämpfer et al. 2012; Oliveira et al. 2009; Woo et al. 2014). *Xanthomonas* and *Pseudoxanthomonas* showed none of the three ligninolytic enzymatic activities (Lip, Lac and MnP). Interestingly as found with various hydrolytic activities, the Bacillus strains demonstrated a wide and variable range for this trait (Table 3).

### Decolorization and utilisation of pulping liquors as carbon source by bacteria

Strains were screened for their potential for bioremediation of black, green and white pulping liquors (Table 4). Growth was tested using Mm agar plates supplemented with pulp liquors as the sole carbon source (5 % v/v) at both pH 7.0 (data not shown) and pH 12.0, and temperature of 37 °C as previously described (Negrão et al. 2015). Thirty-two strains grew at pH 12.0 within 48 h on Mm medium containing pulping liquor(s) as sole carbon source (Chandra et al. 2011; Daizong et al. 2014; Wesenberg et al. 2003).

**Table 4:** Detection of metabolic pathways with potential industrial applications

Strains	Dyes decolorization (pH7)				Mm + 5 % liquors (pH12)			PHA synthesis (pH7)	
	MB	RBB	MG	CSB	Black	Green	White	Sudan black	Nile blue
PS1	D	D	+^a^	W	+^a^	+	−	P^+^	P^+^
PS2	+^a^	−	−	+^a^	+^a^	+	−	−	−
PS3	D	−	−	D	−	+	+	w	P^−^
PS4	D	+^a^	−	+^a^	+^a^	+	+	P^+^	P^+^
PS4.2	D	D	+^a^	+	+^a^	−	+	w^−^	P^+^
PS5	D	−	D	+^a^	−	+	+	P^+^	P^−^
PS6	+^a,e^	+^a,e^	−	+^a^	+	+	+	−	−
PS7	D	+^a,e^	+^a^	+^a^	+	+	−	P^+^	P^+^
PS8	D	+^a^	−	−	+^a^	+	+	P^+^	P^+^
PS9	+^a^	−	−	+^a^	+^a^	−	−	P^+^	P^+^
PS10	D	−	−	+^a^	+	−	−	P^+^	P^+^
PS11	D	−	−	+^a^	+^a^	+	−	P^+^	P^+^
PS12	D	D	−	+^a^	−	+	−	P^+^	P^−^
PS13	+^a^	−	−	+^a^	+	+	+	P^+^	P^+^
PS14	D	+^a^	−	−	+	+	−	P^+^	P^+^
PS15	+^a^	−	−	+^a^	−	−	−	P^+^	P^+^
PS16	+^a^	D	−	+^a^	+^a^	+−	−	P^+^	P^+^
PS17	+^a^	−	−	D	−	−	−	−	−
PS18	D	−	−	−	+	−	+	P^+^	P^+^
PS19	+^a^	−	−	−	+^a^	−	−	P^+^	P^−^
PS20	D	D	−	D	−	−	−	P^+^	P^+^
PS21	D	−	+^a^	+^a^	+^a^	+	−	w	P^−^
PS22	D	D	−	−	−	−	−	w	w
MS1	D	W	+^a^	D	+	−	−	P^+^	P^+^
MS2	D	D	−	−	−	−	+	−	−
MS3	D	+^a^	+	D	+^a^	+	+	P^−^	P^−^
MS4	D	D	+^a^	−	+	+	−	−	−
MS5	−	+	−	D	+	−	−	P^+^	P^+^
MS6	D	+^a,e^	W^a^	+^a^	−	+	+	−	−
MS7	+^a^	+^a^	+^a,e^	+^a^	−	+	+	P^+^	P^+^
MS8	+^a^	+^a^	−	D	+^a^	+	−	−	−
MS9	D	−	−	D	−	+	−	P^+^	P^+^
MS10	w^a^	+^a^	D	D	−	+	−	−	−
MS11	w^a^	+^a^	D	D	+^a^	+	−	P^+^	P^+^
IS1	+^a^	−	−	D	−	−	−	P^+^	P^+^
IS2	D	−	W^a^	+^a,e^	+^a^	−	−	−	−
IS3	D	−	−	+	+^a^	−	+	P^+^	P^+^
IS4	+^a^	−	−	+^a^	−	−	−	w	w^−^
IIS1	+^a^	D	−	+^a^	−	−	−	P^+^	P^+^
IIS2	D	+^a^	D	D	−	−	−	P^+^	P^+^
IIS3	+^a^	+^a,e^	−	D	−	−	−	−	−

Superscript letter a: absorption; e: elimination

+, growth; −, no growth; w, weak growth; P^+^, PHA positive; P^−^, PHA negative; w, weak production of PHA; D, dyes degradation; RBB, Remazol Brilliant Blue; MG, Methyl Green; MB, Methyl Blue; CSB, Chicago Sky Blue

Twenty-four strains grew and decolorized medium containing black liquor. Black liquor contains a high content of lignin (>40 %) (Huang et al. 2007; Schallmey et al. 2004; Negrão et al. 2015), in addition to organic acids, and various polysaccharide degradation by-products (Martínez et al. 2005; Mathews et al. 2013). Among this group, fifteen strains in the genera *Pantoea, Paenibacillus, Bacillus, Citrobacter, Klebsiella, Pneumonia* and *Aeromonas* demonstrated the ability to adsorb the black color from the agar medium within 24 h, and eventually decolorize the compounds responsible for its dark colour (Table 4). This absorption/degradation phenomenon is similar to that reported by Bandounas et al. (2011). These strains were predominantly isolated from IS, PS and MS sludge.

Twenty-two strains grew on Mm medium supplemented with 5 % green liquor (pH 12.0) within 24 h (Table 4). This liquor is a poor source for lignin (Van Dyke 2004). Certain strains (*Paenibacillus* PS12, *Raoultella* MS9 and *Serratia* MS10) grew on the green liquor but not on black or white liquors, suggesting that these strains are either adapted to a lower lignin content than the one found in black liquor or are inhibited by other compounds present in the black liquor.

White liquor is the poorest source of carbon among the three liquors, consisting primarily of inorganics (Na_2_S, NaOH and Na_2_CO_3_). Only thirteen strains belonging to *Paenibacillus* strains PS3 and PS4, *Klebsiella* strains PS4.2, PS5, PS6, PS8, PS13 and PS18, *Bacillus* IS3, *Xanthomonas* MS2, *Pantoea* MS3, *Serratia* MS6 and *Pseudoxanthomonas* MS7 were able to grow on Mm agar plate supplemented with 5 % white liquor at pH 12.0 (Table 4). Certain strains (MS6, MS7, PS3 and PS5) which were unable to grow and stand high lignin concentrations found in black liquor were able to grow on the other liquors, while nine strains (PS15, PS17, PS20, PS22, IS1, IS4) and all IIS strains were unable to grow on any of the three liquors.

Five strains *Paenibacillus* PS4 and PS6, *Bacillus subtilis* PS8, *Bacillus tequilensis* PS13 and *Bacillus cereus* MS3 were able to grow on all the three liquors, suggesting a high degree of resistance to the compounds present in this material (phenolic compounds, organic and inorganic content) as well as the ability to metabolise components contained within. Bacteria isolated from IIS showed no growth on pulping liquor, while strains originating in IS showed a preference for black liquor whereas those from MS preferred green liquor. Finally, strains isolated from PS showed preference to black liquor and grew on green but had little affinity toward white pulping liquor. Clearly, a consortium of selected sludge bacterial strains might become the spearhead of an efficient lignin degradation strategy. In addition, they may also find application for decreasing color associated with black liquor, enhancing a number of paper mills’ products.

### Decolorization of industrial dyes by sludge bacteria

**Fig. 1:**
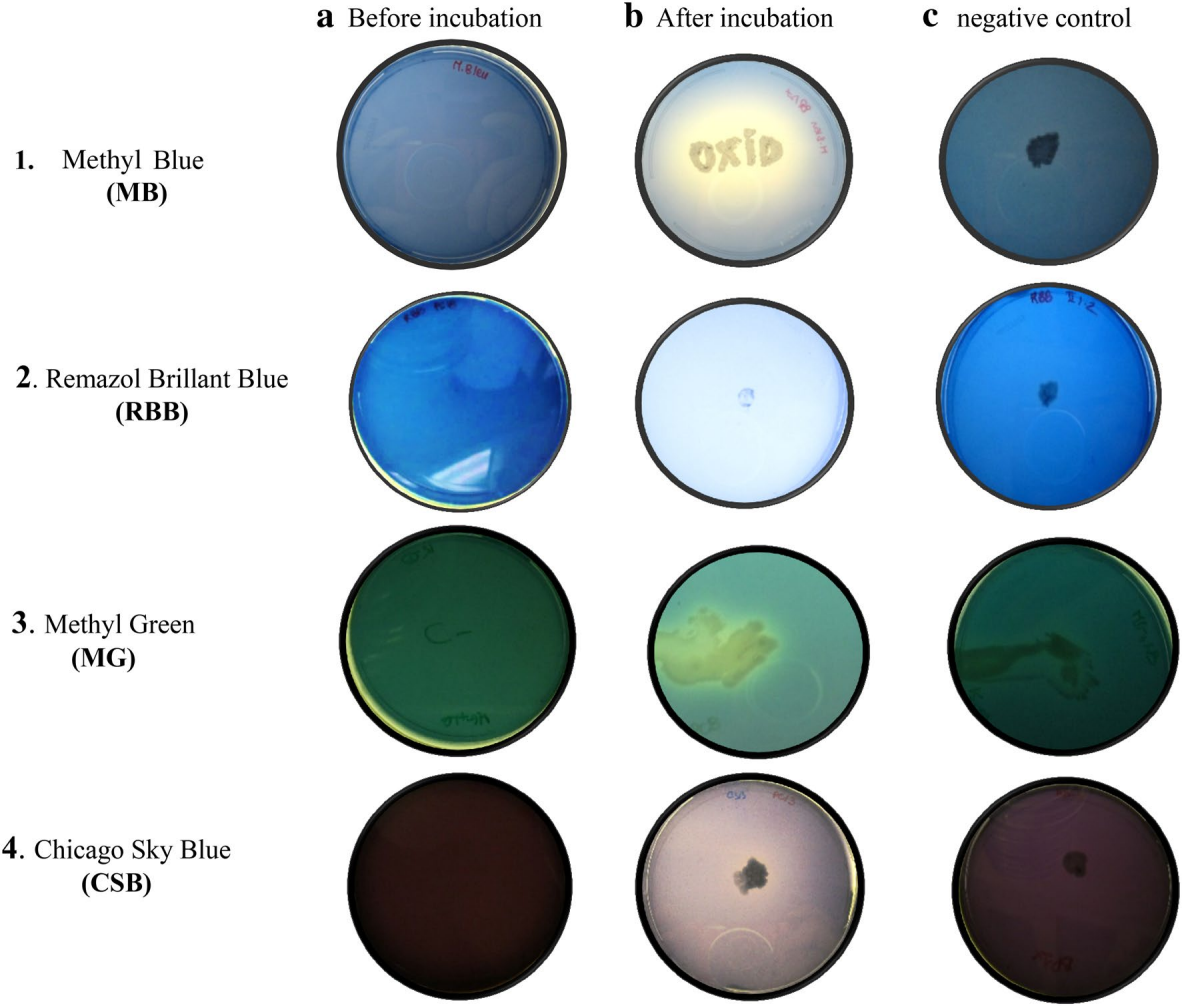
Examples of isolated bacterial decolorization of dye-containing plates. Decolorization of 0.05 % dye-containing plates (1.MB, 2.RBB, 3.MG and 4.CSB) after 24 h incubation at 37 °C and pH7

In order to address the use of these strain for potential use in bioremediation of industrial dyes we screened them on solid medium containing four different ligninolytic indicator dyes or synthetic lignin-mimicking dyes (0.05 % w/v): Methyl blue (MB), Chicago sky blue (CSB), Methyl green (MG) and Remazol brillant blue (RBB; see Fig. 1). Ten strains decolourized all four dyes (Table 4). Among these *Paenibacillus* and *Bacillus* strains were the most efficient, decolourizing the dyes after only 24 h incubation (Table 4), in common with recently described abilities in related strains. The ability to degrade these dyes was not limited to specific genera, and various strains of *Bacillus, Paenibacillus,* and *Klebsiella* had this capability. Methyl blue (MB) and Chicago sky blue (CSB) are the most similar to lignin, and were decolorized by the most of the strains examined (Table 4). Much fewer strains were able to decolorize RBB and MG dye (twenty-two and fourteen strains respectively; Table 4). Furthermore, there was no strict correlation between the ability to decolorize industrial dyes and to grow on pulping liquors, suggesting that different pathways control these characteristics.

**Fig. 2:**
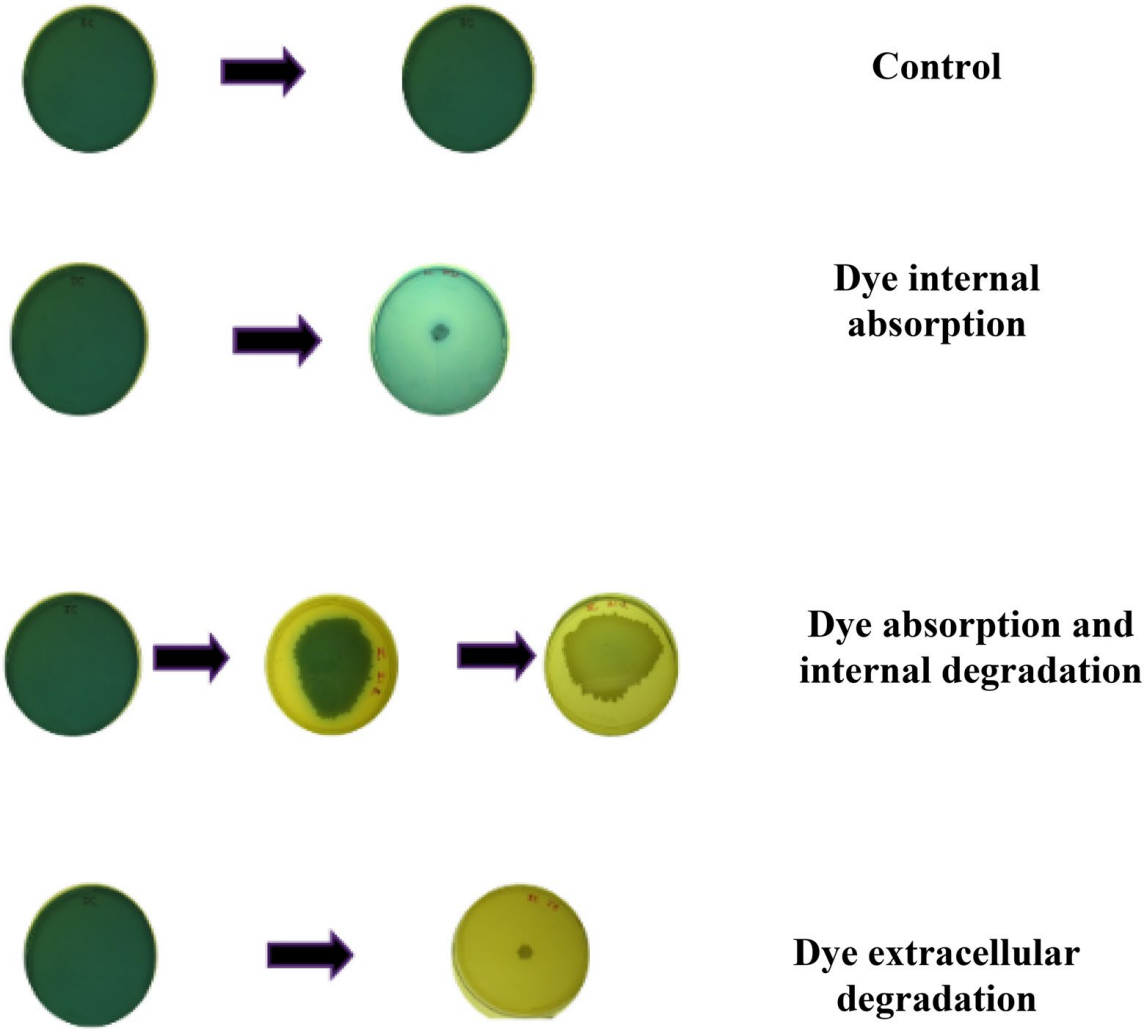
Mechanisms of bacterial degradation of dye-containing plates

In fungi, decolorization of dye proceeds via three different mechanisms, as recently described by Blanca et al. (2007). These include (1) absorption and concentration of the dye (2) intracellular absorption and subsequent degradation of the dye and (3) extracellular degradation of dye. We observed similar mechanisms to decolourize these dyes with our strains (Fig. 2).

This previous study showed that the dyes [they used dyes like RS (red) and 10B (blue)] could bind to the cells and then absorbed inside them. After intracellular compartmentation the dye could be eliminated. It was proposed that some proteins could play a role considering that such dyes are also known to bind to proteinaceous materials (Sahoo and Gupta 2005). In addition, the presence of an intact membrane improved the binding for most dyes. Thus, membrane proteins, intracellular compartments and metabolic abilities may have an impact on dye binding and elimination, leading to different scenarios observed here.

### Bioplastic production by sludge microflora

**Fig. 3:**
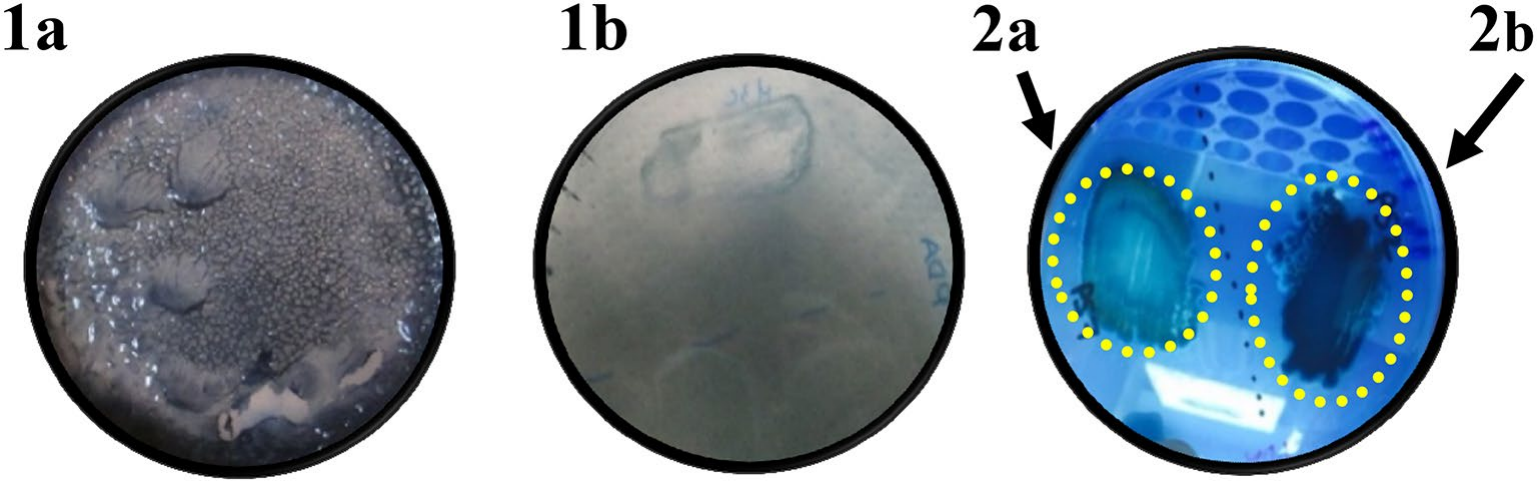
Detection of bacteria producing PHA after Sudan Black B (**1a**, **1b**) and Nile blue A (**2a**, **2b**) staining. **1a** PHA positive bacteria revealed with Sudan Black B staining. **1b** PHA negative bacteria revealed with Sudan Black B staining. **2a** PHA negative with Nile blue staining. **2b** PHA positive with Nile blue staining

Industrial waste like Kraft paper mill sludge may represent a potential source of feedstock used for bioplastic (PHA) production (Priest 1977; Satoh et al. 1999). Twenty-one of the isolated strains produced PHA as determined by staining with Sudan black B and Nile blue A (Fig. 3), and the best producers included *Klebsiella, Bacillus, Paenibacillus, Citrobacter, Serratia, Pseudoxanthomonas* and *Aeromonas* (Table 4). Ten bacterial strains did not grow under our test conditions (PD agar, pH 7.0 and 37 °C) or produce any PHA (Table 4). Among all strains, those within the genera *Bacillus* and *Paenibacillus* showed the greatest degree of staining and may hold the best potential for production of PHA. *Klebsiella is* also known to produce PHA (Bandounas et al. 2011) and unlike the latter species does not form spores which could potentially make the extraction of intracellular PHA easier (Verlinden et al. 2007). As far as we are aware, *B. amyloliquefaciens* PS1 is the first strain of this species shown to produce PHA.

## Conclusion

Bacteria represent an almost inexhaustible source for different industrial enzymes and offer advantages over other sources due to their rapid growth, moderate production costs, breath of enzyme complexity, and biodiversity. Here, we have examined the readily cultivable bacterial community contained in Kraft paper mill sludge and assessed the potential of theses isolates for industrial enzymatic applications. Microbial diversity and enzyme secretion was partially dependent on the sludge composition, where certain genera were found to be specific to the type of sludge sampled. For example *Serratia* and *Raoultella* species were found only in MS, whereas species of *Rahnella* only in IS. Press sludge (PS) hosted the most diverse bacterial community, with many of these isolates tolerating extremes in both temperature and pH conditions (pH 12). In addition, PS strains also expressed the largest number of hydrolytic and ligninolytic enzymatic activities, a possible consequence of the dense and highly complex composition of PS sludge. Similarly, the bacterial community found in machine sludge (MS) was also quite diverse, although these isolates were devoid of xylanase activity, which may reflect the scarcity of xylan fibers in this type of sludge. A diverse range of *Bacillus* species encompassing a rich variety of enzymatic activities were associated with the four sludge samples used in this study. Most sludge types contained strains with ligninolytic activities, and the ability to decolourize lignin-mimicking dyes. Many of the isolated strains also grew on media containing Kraft pulping liquor as the sole carbon source. Regardless of the large difference in sludge composition, all strains had the ability to thrive in the presence of lignin or dyes mimicking lignin, indicating that this ability is fundamental for survival in such an environment. Finally, this study clearly demonstrates that bacteria found in a Kraft paper mill have a significant potential for use in industrial applications including bioplastics, stain control and bioremediation.

## Additional file

10.1186/s40064-016-3147-8 Morphological characterization of bacterial strains screened from different paper mill sludges. **Table S2**. Growth temperature and pH of bacterial strains isolated from the paper mill sludges.

## Footnotes

1 http://www.prnewswire.com/news-releases/global-markets-for-enzymes-in-industrial-applications-266011071.html.
