# Supplementary material for: Microbial diversity in various types of paper mill sludge: identification of enzyme activities with potential industrial applications
Source: Springerplus. 2016 Sep 6;5(1):1492. doi: 10.1186/s40064-016-3147-8 (PMC5013001; doi:10.1186/s40064-016-3147-8)
Supplement: Supplementary file 1 — 10.1186/s40064-016-3147-8 Morphological characterization of bacterial strains screened from different paper mill sludges. Table S2. Growth temperature and pH of bacterial strains isolated from the paper mill sludges. [file 40064_2016_3147_MOESM1_ESM.docx]

**Supplementary material**

*Supplemtary materials*

**Table S1**. Morphological characterization of bacterial strains screened from different paper mill sludges.

| **Strains** | **Gram** | **Oxy** | **Cat** | **Bacillus/**  **Coccus** | **Spores** | **Form** | **Elevation** | **Shape** | **Size** | **Surface** | **Opacity** | **Texture** | **Color** | **Smell** |
| --- | --- | --- | --- | --- | --- | --- | --- | --- | --- | --- | --- | --- | --- | --- |
| **PS1** | + | + | + | bacillus | + | circular | high | irregular | big | rough | opaque | dry | cream | average |
| **PS2** | - | + | + | coccus | - | circular stretched | flat | irregular | average | smooth | opaque | frothy | cream | average |
| **PS3** | + | + | + | bacillus | + | circular | high | regular | average | frothy | not shiny | dry | white | strong |
| **PS4** | + | + | + | bacillus | + | circular stretched | little high | irregular | average | frothy | not shiny | dry | white | strong |
| **PS4.2** | - | + | + | coccus | - | circular | high | regular | big | smooth | shiny | creamy | dark brown | strong |
| **PS5** | + | - | + | bacillus | + | stretched | little high | irregular | big | dry wrinkled | opaque | dry | white | strong |
| **PS6** | + | + | + | bacillus | + | circular | high | regular | average | frothy | not shiny | dry | white | strong |
| **PS7** | + | + | + | bacillus | + | circular | high | irregular | big | frothy | not shiny | dry | white | strong |
| **PS8** | + | + | + | bacillus | + | circular stretched | high | regular | small | smooth | shiny | creamy | cream | average |
| **PS9** | + | + | + | bacillus | + | circular stretched | little high | irregular | average | frothy | not shiny | dry | white | strong |
| **PS10** | + | + | + | bacillus | + | stretched | flat | irregular | average | rough | not shiny | rough | white | strong |
| **PS11** | + | + | + | bacillus | + | circular | high | regular | average | smooth | opaque | frothy | white | light |
| **PS12** | + | + | + | bacillus | + | circular stretched | flat | irregular | average | rough | not shiny | frothy | white | strong |
| **PS13** | + | + | + | bacillus | + | stretched | flat | irregular | average | rough | not shiny | creamy | white | strong |
| **PS14** | + | + | + | bacillus | + | circular stretched | high | irregular | average | rough | not shiny | dry wrinkled | white | strong |
| **PS15** | + | - | + | bacillus | + | circular stretched | high | irregular | average | rough | opaque | granular | cream | low |
| **PS16** | - | + | + | bacillus | - | circular stretched | high | irregular | average | rough | opaque | granular | cream | strong |
| **PS17** | + | + | + | bacillus | + | circular | high | regular | small | smooth | shiny | smooth creamy | transparent | light |
| **PS18** | + | + | + | bacillus | + | circular | flat | irregular | average | rough | opaque | granular | cream | strong |
| **PS19** | + | - | + | bacillus | - | circular stretched | flat | irregular | average | rough | opaque | granular | cream | strong |
| **PS20** | + | + | + | bacillus | + | stretched | convex | irregular | average | rough | not shiny | dry wrinkled | White creamy | strong |
| **PS21** | + | - | + | bacillus | + | circular stretched | flat | irregularirregular | big | rough | opaque | granular | White creamy | strong |
| **PS22** | + | - | + | bacillus | + | circular | high convex | regular | average | rough | opaque | dry wrinkled | White creamy | light |
| **MS1** | - | + | - | bacillus | - | circular | high | regular | average | smooth | opaque | shiny | cream | strong |
| **MS2** | + | + | + | bacillus | + | circular | convex | regular | small | smooth | shiny | creamy | cream green | strong |
| **MS3** | - | + | + | coccus | + | circular | high convex | regular | small | smooth | shiny | creamy | cream | light |
| **MS4** | + | - | + | bacillus | + | circular | high | regular | average | smooth | shiny | creamy | cream | strong |
| **MS5** | - | - | + | coccus | + | circular | convex high | regular | small | smooth | shiny | bright | transparent | light |
| **MS6** | - | + | + | coccus | + | circular | high | regular | average | smooth | shiny | creamy | dark cream | light |
| **MS7** | - | - | + | coccus | + | circular | high convex | regular | very small | smooth | very shiny | creamy | transparent | strong |
| **MS8** | - | - | + | coccus | + | circular | high convex | regular | small | smooth | bright shiny | creamy | transparent | light |
| **MS9** | - | - | + | coccus | + | circular | high | regular | small | smooth | shiny | creamy | transparent | light |
| **MS10** | + | + | + | coccus | + | circular | high | regular | average | smooth | shiny | creamy | white cream | light |
| **MS11** | + | - | + | bacillus | + | circular | convex | regular | average | smooth | shiny | creamy | cream | strong |
| **IS1** | + | + | + | bacillus | - | circular | little high | regular | small | smooth | opaque | creamy | cream | low |
| **IS2** | - | + | + | coccus | - | circular | high | irregular | big | wrinkled | opaque | dry | cream | strong |
| **IS3** | + | + | + | bacillus | + | circular | high convex | regular | small | smooth | shiny | creamy | transparent | light |
| **IS4** | + | + | + | bacillus | + | circular | high | regular | small | smooth viscous | shiny | bright shiny | cream | strong |
| **IIS1** | - | + | - | bacillus | + | circular | high | regular | small | smooth | bright shiny | creamy | clear cream | strong |
| **IIS2** | + | + | - | bacillus | + | circular | high | regular | small | smooth | shiny | viscous creamy | cream | strong |
| **IIS3** | - | + | + | coccus | + | circular | high | regular | small | smooth | bright shiny | creamy | clear cream | light |

***Oxy: oxidase, Cat: catalase***

**Table S2.** Growth temperature and pH of bacterial strains isolated from the paper mill sludges.

| **Strains** | **37 °C** | **50°C** |
| --- | --- | --- |
| **PS1** | **+ ^6-10^** | **+^6-10^** |
| **PS2** | **+^7-10^** | **+^7-10^** |
| **PS3** | **+^6-10^** | **+^6-10^** |
| **PS4** | **+^6-10^** | **+^7-10^** |
| **PS4.2** | **+^6-10^** | **+^6-10^** |
| **PS5** | **+^6-10^** | **+^6-7^** |
| **PS6** | **+^6-10^** | **+^7-10^** |
| **PS7** | **+^7-10^** | **+^7^** |
| **PS8** | **+^6-10^** | **+^6-10^** |
| **PS9** | **+^7-10^** | **+^7-10^** |
| **PS10** | **+^6-10^** | **+^7-10^** |
| **PS11** | **+^6-10^** | **+^7-10^** |
| **PS12** | **+^7-10^** | **+^7^** |
| **PS13** | **+^6-10^** | **+^7-10^** |
| **PS14** | **+^6-10^** | **+^7-10^** |
| **PS15** | **+^6-10^** | **+^7^** |
| **PS16** | **+^6-10^** | **+^6-7^** |
| **PS17** | **+^6-10^** | **+^7-10^** |
| **PS18** | **+^6-10^** | **+^6-10^** |
| **PS19** | **+^7-10^** | **+^7-10^** |
| **PS20** | **+^7-10^** | **+^7-10^** |
| **PS21** | **+^6-10^** | **+^7-10^** |
| **PS22** | **+^6-10^** | **+^6-10^** |
| **MS1** | **+^6-10^** | **+^6-7^** |
| **MS2** | **+^6-10^** | **+^6-10^** |
| **MS3** | **+^7-10^** | **-** |
| **MS4** | **+^7-10^** | **-** |
| **MS5** | **+^7-10^** | **-** |
| **MS6** | **+^6-10^** | **-** |
| **MS7** | **+^6-10^** | **-** |
| **MS8** | **+^7-10^** | **-** |
| **MS9** | **+^7^** | **-** |
| **MS10** | **+^7^** | **-** |
| **MS11** | **+ ^7-10^** | **-** |
| **IS1** | **+^7^** | **-** |
| **IS2** | **+^7-10^** | **-** |
| **IS3** | **+^7^** | **-** |
| **IS4** | **+^7^** | **-** |
| **IIS1** | **+^7^** | **-** |
| **IIS2** | **+^7^** | **-** |
| **IIS3** | **+^7^** | **-** |

***+****: Growth,* ***-****: No growth, Superscript numbers show growth pH range*
